# Supplementary material for: Identification of ZmBK2 Gene Variation Involved in Regulating Maize Brittleness
Source: Genes (Basel). 2023 May 23;14(6):1126. doi: 10.3390/genes14061126 (PMC10298650; doi:10.3390/genes14061126)
Supplement: Supplementary file 1 [file genes-14-01126-s001.zip › Supplementary Materials Figure S1-S7.pdf]

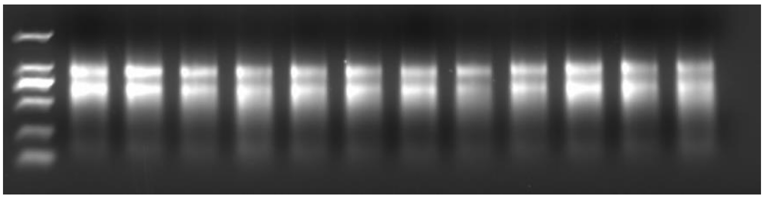

Figure S1: Electrophoresis image of 12 RNA sample bands.

Q-Zm00001eb432200 Q-Zm00001eb399710

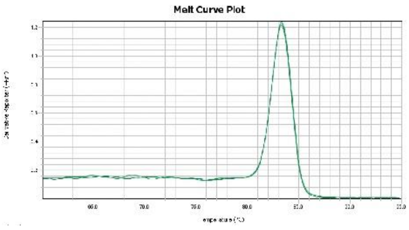

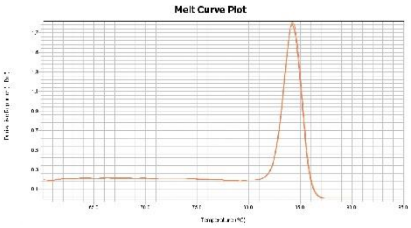

Q-Zm00001eb036670 Q-Zm00001eb280690

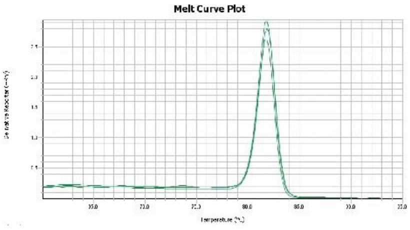

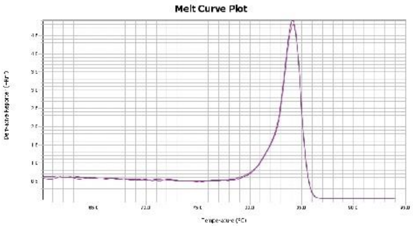

Q-Zm00001eb002620 Q-Zm00001eb327820

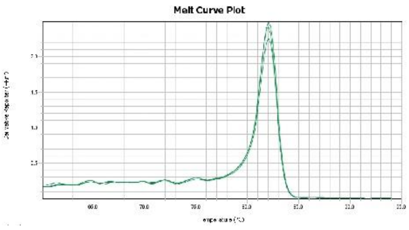

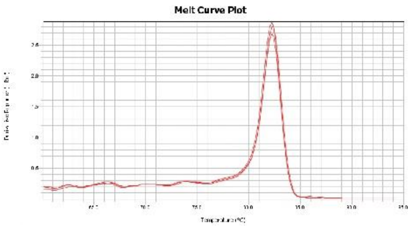

Q-Zm00001eb122830 Q-Zm00001eb174480

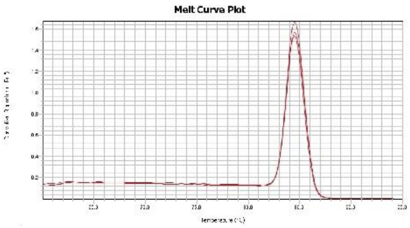

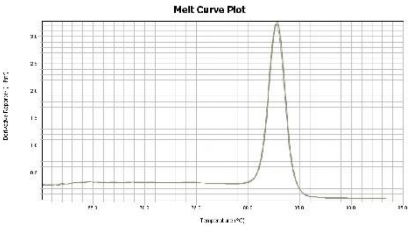

Q-Zm00001eb349960 Q-Zm00001eb414390

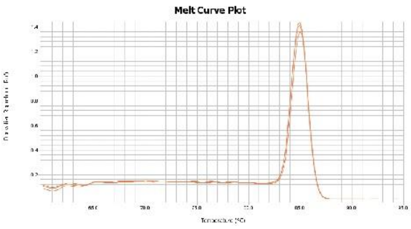

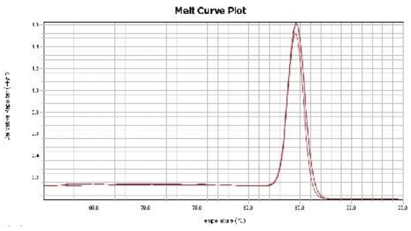

Q-Zm00001eb082890 Q-Zm00001eb244670

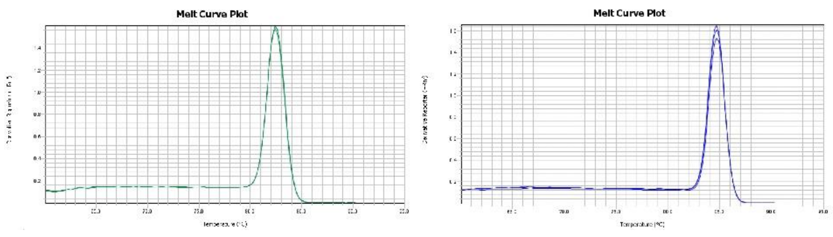

Q-Zm00001eb022710 Q-Zm00001eb282430

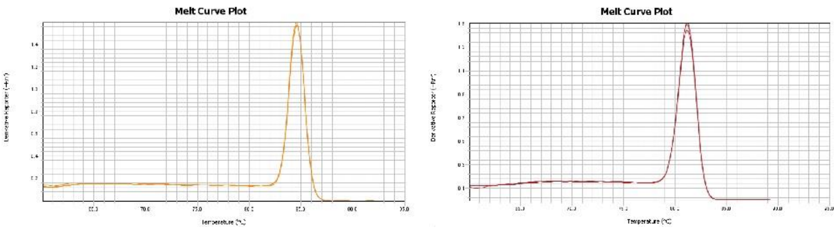

Q-Zm00001eb282430 Q-Zm00001eb393680

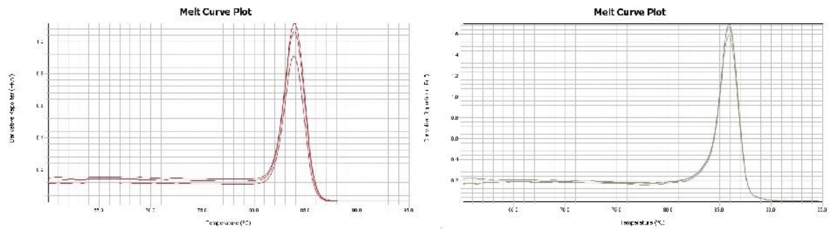

QZmACTIN Q-ZmBK2

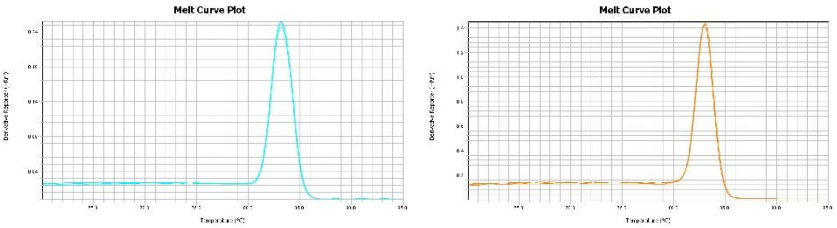

Figure S2: Melting Curve Analysis 12 pairs of primers.

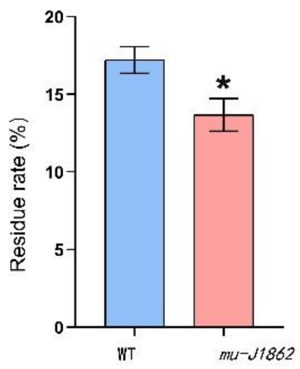

Figure S3: Determination of residue rate in *bk2* and wild type 24 days after pollination. Each group was measured using 100g maize kernels. The error bars were obtained from three independent measurements. (* and ** represent t-test at $P < 0.05$ and $P < 0.01$, respectively).

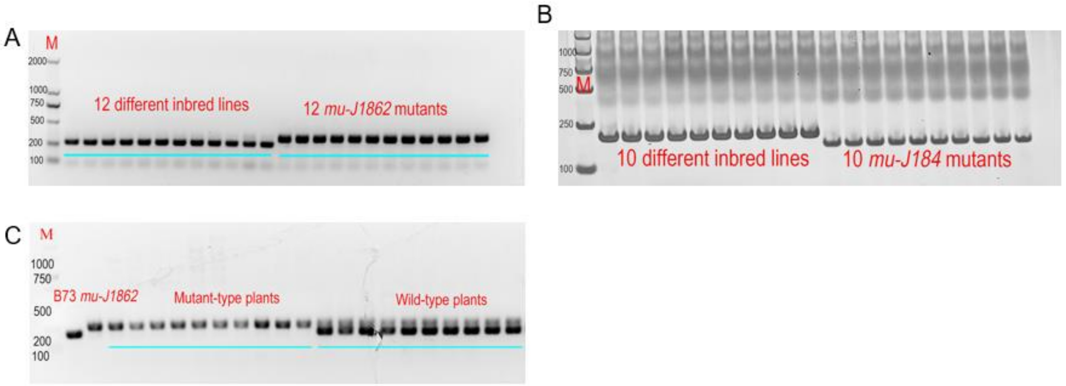

Figure S4: *BK2-J1862F/R* and *BK2-J184F/R* were used as functional markers to detect the brittle phenotype. (A) *BK2-J1862F/R* was used to distinguish *mu-J1862* and different inbred lines. (B) *BK2-J184F/R* was used to distinguish *mu-J184* and different inbred lines. (C) Verification of *BK2-J1862F/R* marker in (*mu-J1862*/B73)/*mu-J1862* backcross population.

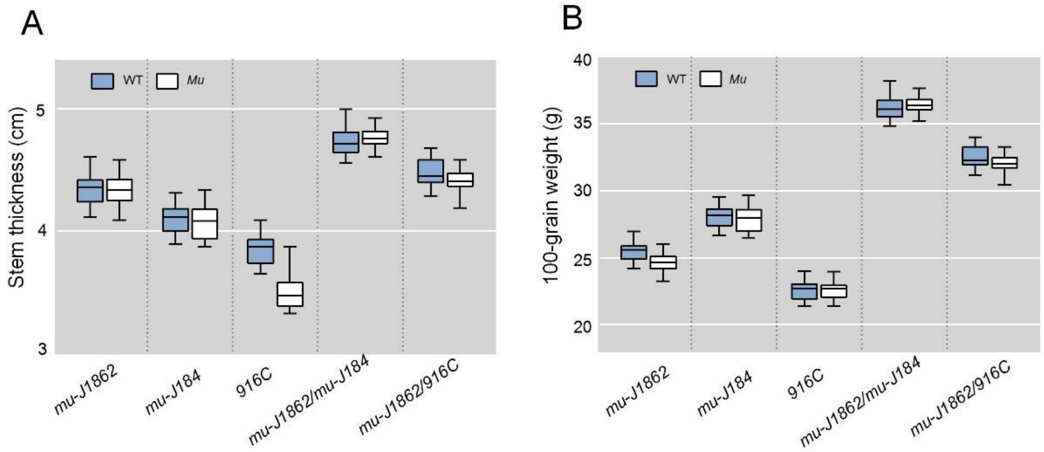

Figure S5: Mutant stalk thickness and 100-grain weight. (A) Mutant stalk thickness. (B) Mutant 100-grain weight. Data were obtained from 25 individuals for each plant line at maturity. The values are presented as means ± SD and error bars were obtained from three independent measurements.

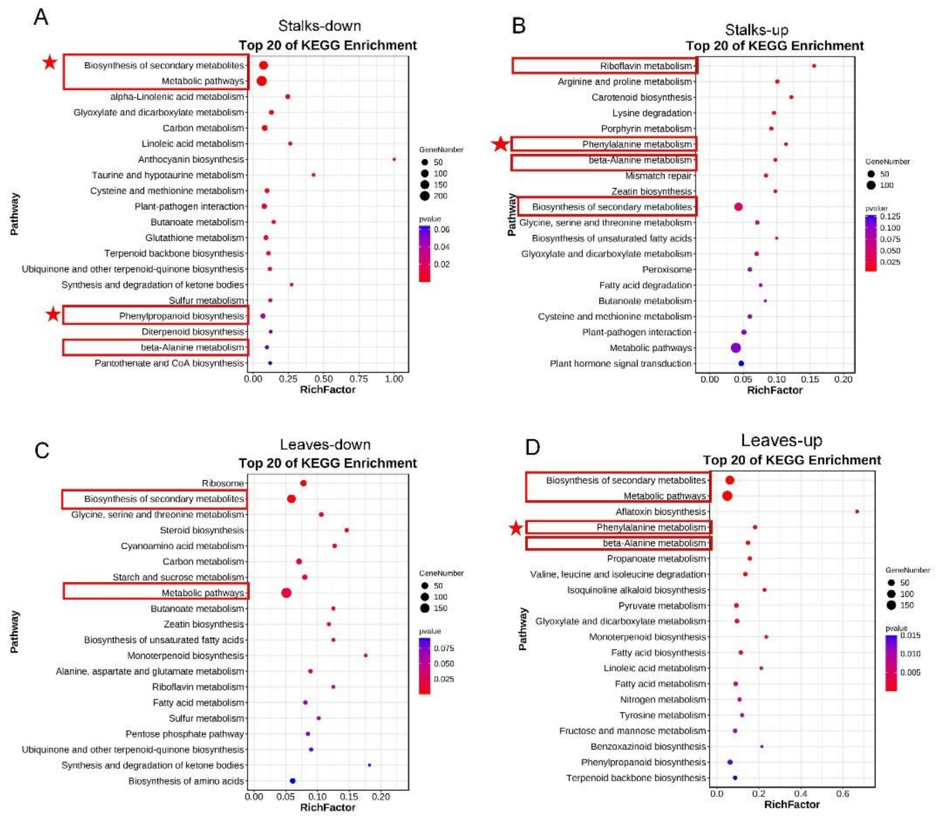

Figure S6 Enrichment analysis of up-regulated and down-regulated genes in mutant leaves and stalks.

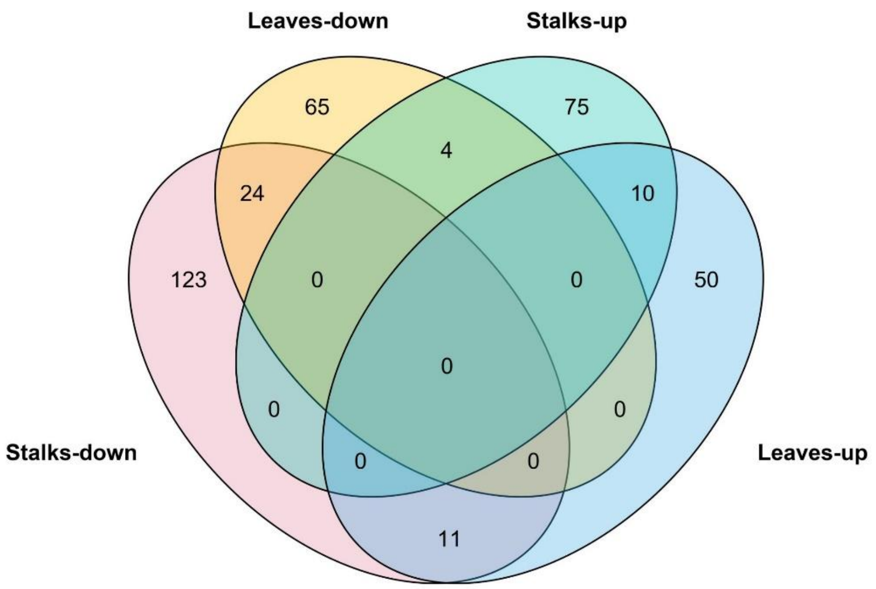

Figure S7: Venn diagram of total TFs (Padj < 0.01) among genotypes.
