# Supplementary material for: Identification of ZmBK2 Gene Variation Involved in Regulating Maize Brittleness
Source: Genes (Basel). 2023 May 23;14(6):1126. doi: 10.3390/genes14061126 (PMC10298650; doi:10.3390/genes14061126)
Supplement: Supplementary file 1 [file genes-14-01126-s001.zip › Supplementary Materials Table S1-S4ú1⁄4Table S6-S7.pdf]

Table S1: RIN numbers of RNA samples.

RNA Samples	RIN
WT-Stalks-1	9.90
WT-Stalks-2	9.90
WT-Stalks-3	9.80
Mu- Stalks -1	10.00
Mu- Stalks -2	9.80
Mu- Stalks -3	10.00
WT-Leaves-1	8.7
WT- Leaves-2	9.4
WT- Leaves-3	9.3
Mu- Leaves-1	9.3
Mu- Leaves-2	9.4
Mu- Leaves-3	9.2

Table S2: RT-qPCR primer information.

Q-ZmBK2-F	AAGGACCTTCACCTTCAGCA
Q-ZmBK2-R	GGCAGGTAGGGGTAGGAGTC
Q-Zm00001eb432200-F	CGACGAGAACAGCATCCTC
Q-Zm00001eb432200-R	GCGCCTGTAGGAAGGACTC
Q-Zm00001eb399710-F	ACTGCTAGGGTCCAAAGTTTAA
Q-Zm00001eb399710-R	CCAATCATAACCCCAGTGTTTG
Q-Zm00001eb036670-F	ACTGCTAGGGTCCAAAGTTTAA
Q-Zm00001eb036670-R	CCAATCATAACCCCAGTGTTTG
Q-Zm00001eb280690-F	CCCACCACCTACATCAACG
Q-Zm00001eb280690-R	ACTGCAAAACAAATAGCACGAG
Q-Zm00001eb002620-F	TAGAATCCGCCTCTTTACTGAC
Q-Zm00001eb002620-R	GCAAATAAGTTCTTTGTCGGGT
Q-Zm00001eb327820-F	AATTCAAGCCCTATCCTCTCAG
Q-Zm00001eb327820-R	CTAGCTAGATACGTACGTCCAC
Q-Zm00001eb122830-F	AAGTCACCCATCACCATACAG
Q-Zm00001eb122830-R	TGCATCTCTTGTGGTTATAGCA
Q-Zm00001eb174480-F	GACTCCTTCGTTGGCTTCTTC
Q-Zm00001eb174480-R	GCAGAATGGCATGGAAAGATAG
Q-Zm00001eb349960-F	GAGGCTGGCGATCAATCTAC
Q-Zm00001eb349960-R	GATTCAAACGATCCGAAAGG
Q-Zm00001eb414390-F	GATTCCCAGAAAGCTCGATCTA
Q-Zm00001eb414390-R	CCGTGTTGAATTTGTCGTAGAG
Q-Zm00001eb082890-F	GATGAGCAGGACTGGGTGTT
Q-Zm00001eb082890-R	TGCCAGAGCTTGTATTGCAC
Q-Zm00001eb244670-F	AGACAGTCCACTCCAGTATTTG

Q-Zm00001eb244670-R	AGTGAGTCTGGTCTTGGTTTAG
Q-Zm00001eb022710-F	CACCACTACGCCATCATCTG
Q-Zm00001eb022710-R	CTTCCTCTCGTACCGCCTGA
Q-Zm00001eb282430-F	GTGTGGTATACTGGTAACCCAT
Q-Zm00001eb282430-R	AATTAACCGTCCCACTACTAGC
Q-Zm00001eb393680-F	GAAGGCGTCGTACAGGGATAG
Q-Zm00001eb393680-R	CTTGATCCTCTCGATGACGTC
QZmACTIN-F	TACGAGATGCCTGATGGTCAGGTCA
QZmACTIN-R	TGGAGTTGTACGTGGCCTCATGGAC

Table S3: Molecular markers for map-based cloning.

Xwd2-28-F	TAAGTGCCCGTGGGCATGA
Xwd2-28-R	GGATCCGTCCGAGTGTACC
Xwd1-19-F	GAGTAGATTCCTCCTCAGTCGC
Xwd1-19-R	GCTAACTAAGTTGGTCTGGTGC
Xwd1-30-F	GGAACATGCAACTATAGAGGTGGC
Xwd1-30-R	AAAGAGGGAGCATTTGCCG
Xwd3-38-F	GCAACGATGGAGGCACAG
Xwd3-38-R	TCGCCCCTTTAAACCACGTG
Xwd3-11-F	CGGTCTCCTTCGCAGCA
Xwd3-11-R	CCTTCCTCCCTGATACACCGA
Xwd1-78-F	GGTGGGAGGCTCAAGTGTAA
Xwd1-78-R	TGCGACTCAGGACTCTCGTA
Xwd1-80-F	TGCTCCTCGTTCAACTCTGA
Xwd1-80-R	TCTTCTCCTTTGCCATGAGG
Xwd1-87-F	GGGTTTTCCTGGTTATGTGG
Xwd1-87-R	GGCCCCTTTTGCATAACTCT
Xwd4-21-F	TCTGAGTGAGCCCGAAAGAT
Xwd4-21-R	TCAGGGTCATCCAACAGACA
p-umc1570-F	CAGGAGATGATGAGCGGGAG
p-umc1570-R	GTCGTAGAGGTGGTGCTGCTG
p-umc2341-F	AAACATTTAATCCAACAGCCCAGA
p-umc2341-R	CTGAGCTCCTGATTTCTTGCTCTC
Xwd4-37-F	AAGCTTGCGGAAACTAACGA
Xwd4-37-R	TTTTACGCACTGTGCTCCTG
Xwd6-11-F	GTGAGGCGTCATTTCCTCAT
Xwd6-11-R	AAGAAGTTCACCGCAATGCT
Xwd6-13-F	GTACATGCCCTGCTGGTTTT
Xwd6-13-R	TAGCGTCCCATATCCTCTGG
Xwd3-51-F	GCTTACCCATGACGAAGCAT
Xwd3-51-R	GATGCATTGCCTTCCTTGAT
Xwd3-56-F	AGTTGGCTCGAAGGTGAGAA
Xwd3-56-R	CTCGGCTAACCATCTTCCAA

Xwd6-20-F	CCTAACGCGGTAGAAGTTGC
Xwd6-20-R	GACCCATTCAGCCACAAAGT
Xwd6-21-F	GAAGGAAGGTATCGCAGTGG
Xwd6-21-R	ACCCCTAGCCACCCATCTAC
Xwd7-6-F	ACTTTTCACCGTCGATCACC
Xwd7-6-R	CCGAACCTCGGTAAAACAAA
Xwd3-68-F	GTGCTCTCACAAAGCAACCA
Xwd3-68-R	AAGCGACGTAGCTGTTGGAT
Xwd7-15-F	CGATGGGAAGGAACATCAGT
Xwd7-15-R	GCTGAGCAATCCCACTCTTC
Xwd3-24-F	GATCGGTTAGCCATCTGCAT
Xwd3-24-R	TCAATAACAACCGAGCACCA
Xwd3-13-F	AAGGAGGAGCACAGGGATTT
Xwd3-13-R	GGCACACCGAATAGTCCAGT
Xwd7-16-F	ACGCTTTGTTTGGTGAGCTT
Xwd7-16-R	TCGACAACAAATTGCAGAGC
Xwd7-17-F	TTGCCCGAACCAGTATTCTC
Xwd7-17-R	CCGATACCAGCACACACATC
Xwd3-76-F	CAGGCACAGTTCGAGATTGA
Xwd3-76-R	CGACACTACACCCCAGACCT
BK2-J184F	TTTTGTTTTCCCGTCTCGTC
BK2-J184R	TGCTGATTCACGTTTTGCAT
BK2-J1862F	GACAACGTCACCGAGGTCTT
BK2-J1862R	GCCTCCATGAGAAAGTCGTT

Table S4: The detailed information of raw reads from different samples.

**Sample**	**Raw Data Reads**	**Reads After Filter**	**Clean Reads Rate (%)**	**Mapped Reads**	**Mapped Rate (%)**	**Unique Mapped Reads**	**Unique Mapped Rate (%)**
WT-stalks-1	45569381	43345595	95.12	92.23	39977642	32257959	80.69
WT-stalks-2	46546317	44740319	96.12	91.32	40856860	32983743	80.73
WT-stalks-3	45873901	43254501	94.29	92.38	39958508	31878897	79.78
BK2--stalks-1	46359013	43753636	94.38	92.51	40476489	32968100	81.45
BK2-stalks-2	46182634	44233726	95.78	92.41	40876386	33367394	81.63

BK2-stalks-3	45293237	42906283	94.73	93.14	39962912	33073306	82.76
WT-leaves-1	44982934	42900224	95.37	92.17	39541136	33206646	83.98
WT-leaves-2	47921304	45031649	93.97	90.89	40929266	33795295	82.57
WT-leaves-3	46392308	43808256	94.43	92.38	40470067	33140938	81.89
BK2-leaves-1	43992091	41207391	93.67	91.69	37783057	31397720	83.1
BK2-leaves-2	46994340	44846698	95.43	92.77	41604282	34186238	82.17
BK2-leaves-3	46293481	43816779	94.65	91.45	40070445	32008271	79.88

Table S5: Homologous genes expression of *ZmBK2*.

	WT-Stalks(fpkm)	Mu-Stalks (fpkm)	WT-Leaves(fpkm)	Mu-Leaves(fpkm)
ZmBK2	0.106029333	4.51451	6.16657	1.191805333
Zm00001eb432200	46.34756233	18.0285935	2.822005333	1.289322333
Zm00001eb399710	0.769282	2.192547	0.267863667	0.802857
Zm00001eb036670	1.223054333	2.090569	0.425353333	0.465403333
Zm00001eb280690	9.243331667	18.7463375	0.056700333	0
Zm00001eb002620	9.017306	19.6461675	222.544854	58.607015
Zm00001eb327820	28.58568833	14.0537095	8.971429	3.883357667
Zm00001eb122830	0.088921	0.0141915	33.66627	4.365139333
Zm00001eb174480	34.778782	14.645711	4.217939333	1.733463333
Zm00001eb349960	36.15626467	6.1176735	1.543621333	6.945168
Zm00001eb414390	2.826517667	43.572567	1.400827667	0.850146333
Zm00001eb082890	0.038030333	0.01924	19.46002467	293.281414
Zm00001eb244670	52.75262567	7.435134	4.529734667	0.931244
Zm00001eb022710	15.87407733	53.350956	1.296685333	0.054792667
Zm00001eb282430	0.499068	1.481683	66.26611067	28.34963467
Zm00001eb393680	0.068528	0	160.6773733	12.792574

Table S7: Expression of reciprocal genes were predicted using string.

		WT-Stalks-fpkm	Mu-Stalks-fpkm	WT-Leaves-fpkm	Mu-Leaves-fpkm
GRMZM2G037413 (cesa13)	Zm00001eb153340	0.850608	5.433046	0.058843333	0.347105667
GRMZM2G168364	Zm00001eb317090	41.627244	48.2788465	4.194320333	2.679273
GRMZM2G177242	Zm00001eb123640	139.3360187	96.4099845	0.053545333	0.137356667
GRMZM2G031200	Zm00001eb295810	0.045114333	0.1989885	0	0
GRMZM2G026218	Zm00001eb307070	33.49762967	28.2562395	9.268136667	10.873189
GRMZM2G325907	Zm00001eb416400	0	0.322902	0	0
GRMZM2G097404	Zm00001eb023530	0.052267	0.3888675	0	0.063718667
GRMZM2G147390	Zm00001eb421850	0.263443333	1.7502715	0.078346	0.266753667
